# Differential regulation of interleukin-8 and human beta-defensin 2 in *Pseudomonas aeruginosa*-infected intestinal epithelial cells

**DOI:** 10.1186/s12866-014-0275-6

**Published:** 2014-11-30

**Authors:** Fu-Chen Huang

**Affiliations:** Department of Pediatrics, Kaohsiung Chang Gung Memorial Hospital and Chang Gung University College of Medicine, 123, Ta-pei Road, Niao-sung District, Kaohsiung, Taiwan

**Keywords:** Pseudomonas aeruginosa, Interleukin 8, Human beta-defensin 2, Intestinal epithelia

## Abstract

**Background:**

The human opportunistic pathogen, *Pseudomonas aeruginosa (P. aeruginosa*) carries the highest case fatality rate of all gram-negative infections. Unfortunately, antimicrobial therapy has not been demonstrated to improve clinical outcome and the emergence of multidrug resistant *P. aeruginosa* has become a major concern in the hospital setting. Fever and diarrhea are the two most common initial symptoms in *P. aeruginosa* sepsis in previously healthy infants and children. This implies that intestinal epithelial cells in first contact with the pathogen may play an important role in innate immunity to *P. aeruginosa* infection. Human beta–defensins-2 (hBD-2) and interleukin-8 (IL-8) are crucial for host defense at mucosa but IL-8 may give rise to characteristic pathology of colitis.

**Results:**

*Pseudomonas aeruginosa* strain PAO1 was used to infect SW480, an intestinal epithelial cell. IL-8 and hBD-2 mRNA expression and protein secretion were then assessed in SW480 cells using RT-PCR and enzyme-linked immunosorbent assay (ELISA), respectively. Intracellular signaling pathways and nucleotide-binding oligomerization domain (NOD) 1 protein expression were analyzed by Western blot in SW480 cells in the presence or absence of inhibitors or transfected with siRNA.

We demonstrate that prolonged infection by *P. aeruginosa* results in suppression of IL-8 but enhancement of hBD-2, either protein secretion and mRNA expression, in SW480 cells. Inhibitors of ERK suppressed but inhibitor of PI3K enhanced *P. aeruginosa*-induced IL-8 mRNA expression in SW480 cells while both signaling had no effect on *P. aeruginosa*-induced hBD-2 expression in SW480 cells. On the other hand, NOD 1 was illustrated to get involved in *P. aeruginosa*-induced hBD-2 mRNA expression and protein production in SW480 cells.

**Conclusions:**

The *P. aeruginosa*-induced antimicrobial peptide in IECs continuously protect the host against prolonged infection, while modulation of proinflammatory responses prevents the host from the detrimental effects of overwhelming inflammation. Thus, *P. aeruginosa*-induced innate immunity in IECs represents a host protective mechanism, which may provide new insight into the pathogenesis of inflammatory bowel diseases.

**Electronic supplementary material:**

The online version of this article (doi:10.1186/s12866-014-0275-6) contains supplementary material, which is available to authorized users.

## Background

The human opportunistic pathogen, *Pseudomonas aeruginosa* (*P. aeruginosa*), is a major cause of infection-related mortality among critically ill patients, and carries the highest case fatality rate of all gram-negative infections [1]. Although rare, *P. aeruginosa* bacteremia is often rapidly progressive and can occur with a high mortality rate in previously healthy patients [2,3], even receiving appropriate antimicrobial treatment. Moreover, in a pediatric study of *Pseudomonas* bacteremia, antimicrobial susceptibility was not identified as a prognostic factor [4]. *P. aeruginosa* is not only difficult to treat but also exhibits remarkable ability to acquire resistance to these agents [5]. Overall, resistance rates are on the increase. Multidrug resistance is frequent, and clinical isolates resistant to virtually all anti-pseudomonal agents are increasingly being reported. Therefore, effective immunotherapy may be a useful alternative therapy administered either alone or in combination with antibiotic chemotherapy.

In a study of community-acquired sepsis associated with *P. aeruginosa* in previously healthy infants and children [2], fever and diarrhea were the two most common initial symptoms. *P. aeruginosa* was also isolated in 43% of fecal specimens. This implied that intestinal epithelial cell first contacting the pathogen may play an important role on innate immunity to *P. aeruginosa* infection. In addition to serving as a protective barrier, the epithelium plays an active role in the intestinal immune response through its secretion of inflammatory cytokines, chemokines, and antimicrobial peptides [6,7]. Antimicrobial peptides, such as human β–defensins-2 (hBD-2), are crucial for host defense at mucosal surfaces while chemokines, such as interleukin-8 (IL-8), recruit neutrophils from the circulation into the subepithelial region to defend against the invasion of bacteria, but give rise to characteristic pathology of colitis [8].

Two main families of pattern-recognition receptors involved in innate immune detection have been discovered in human beings. Toll-like receptors (TLRs) are transmembranous molecules [9] and cytosolic Nucleotide-binding oligomerization domains (NODs) [10] are seen as the intracellular counterpart of the TLRs. Both play essential roles in the clearance of *P. aeruginosa* [11]. Shedding of flagellin, recognized by TLR5, from *P. aeruginosa* provokes hBD-2 and IL-8 response in human keratinocytes [12]. Intestinal epithelial cells (IECs) are generally hyporesponsive to extracellular bacterial products, especially TLR2 and TLR4 ligands [13]. The unresponsiveness of IECs to TLR signals sets the stage for the function of NOD proteins as important sensors for the detection of bacteria invading the epithelium [14]. Autophagy plays an essential role in the clearance of *P. aeruginosa* by alveolar macrophages. Two groups of investigators [15,16] have demonstrated that NOD1 and NOD2 are critical for the autophagic response to invasive bacteria because they recruit ATG16L1 to bacterial entry sites at the plasma membrane. Several studies have implicated NOD1-dependent NF-κB activation in the induction of β-defensins and chemokines expression in response to H. pylori [17] and S. flexneri infection [18]. Moreover, a recent study showed that NOD2 is essential in the enhancement of IL-8 induced by S. aureus through activation of c-jun NH2-terminal kinase (JNK) pathway and upregulation of COX2 [19]. The cooperation of TLR5 and NOD2 in IECs regulates inflammatory response to *Salmonella* infection [20].

Therefore, we aim to investigate the intestinal epithelial IL-8 and hBD-2 expression in *P. aeruginosa*-infected IECs and the downstream signaling pathways of TLRs or NODs involved in the effects. Up to now, the innate immunity of IECs to *P. aeruginosa* infection has been completely unknown. We have studied the inflammatory responses in *P. aeruginosa*-infected IECs, and for the first time reveal the differential regulation of *P. aeruginosa*-induced IL-8 and hBD-2 in IECs via PI3K/Akt signal and NOD1 protein respectively. This observation could provide useful information for further understanding of the innate immunity in mucosal *P. aeruginosa* infection.

## Methods

### Cell culture and infection

SW480 and Caco-2 cells (ATCC, Rockville, MD), transformed human colonic epithelial cell lines, were grown in Dulbecco modified Eagle medium (DMEM) supplemented with 10% heat-inactivated fetal calf serum, 100 units/ml penicillin, 100 μg/ml streptomycin sulfate, and 20 mM HEPES (Sigma) in a 5% CO_2_ atmosphere at 37°C. Passage 10–30 will be used for all experiments. For stimulation experiments, cells were seeded in 12-well tissue culture plates (4 cm^2^/well; BD Biosciences) and used at 60–80% confluence.

### Reagents

The PI3K inhibitors, wortmannin and LY294002, and the mitogen-activated protein (MAP) kinase ERK inhibitor, PD98059 were obtained from Calbiochem (San Diego, CA) and stock solutions made in dimethylsulfoxide (DMSO). Standard laboratory reagents were from Sigma (St. Louis, MO, USA) or Fisher Scientific (Pittsburgh, PA, USA).

### Bacterial strain

*P. aeruginosa* strain PAO1 is a well-characterized laboratory strain. It is grown in tryptic soy broth (Difco Laboratories, Detroit, MI) supplemented with 10 μg/ml kanamycin for 6 h until the optical density at 600 nm is 0.5 or the concentration is about 1 × 10^8^ CFU/ml. The bacteria is collected by centrifugation at 3,000 rpm for 10 min, washed twice by resuspension in sterile phosphate-buffered saline (PBS) (GIBCO, Grand Island, NY), and finally suspended at the desired dilution in PBS. Cultured cells were infected at a multiplicity of infection (MOI) of 10 for indicated times.

### Cytokine assays

#### IL-8 ELISA

After treatment, the medium was aspirated at the end of the infection period, from control or infected cells, cleared of any *P. aeruginosa* or cellular debris by centrifugation (5 min, 1,000 × *g*), then stored at —20°C until use. The supernatant medium was collected and IL-8 concentrations determined by enzyme-linked immunosorbent assay (ELISA) as described in detail earlier [20,21].

#### hBD-2 ELISA

After treatment or infection, the supernatant medium of the cultured cells was collected and hBD-2 concentrations determined by enzyme-linked immunosorbent assay (ELISA) as manufacturer’s instructions with some modification. Briefly, 96-well immunoplates were coated with goat anti-hBD-2 antibody (PeproTech, Rocky Hill, NJ). After blocking and washing, 100 μl/well of cell culture supernatants and serial dilutions of standard hBD-2 in cell culture medium were incubated for 30 min at room temperature. Subsequently, wells were incubated with biotinylated goat anti-hBD-2 antibody (Cell Concepts), filled with 50 μl/well of Streptavidin-POD (Roche Diagnostics) and developed by ABTS (Roche Diagnostics). Absorbance is measured at 405 nm with a multichannel photometer (Sunrise; Tecan, Crailsheim, Germany). Because of variations in baseline hBD-2 production, the results are expressed as “fold increase”, representing the normalized hBD-2 produced by infected monolayers divided by the normalized hBD-2 produced by control, uninfected cells.

### Protein extraction

Cytosolic and membranous extracts from untreated and treated SW480 cells were prepared as manufacturer’s instructions with slight modifications [21,22]. Protein concentrations in cell fractions were determined using a Bio-Rad assay kit.

### Western blotting

Equal amounts of total protein were separated by SDS-PAGE and then transferred to nitrocellulose membranes by semi-dry blotting as previously described [23,24]. After blocking the membranes with 5% non-fat dry milk, they were probed with antibodies to either phosphorylated p38, JNK, Akt (Cell Signaling, Beverly, MA), phosphorylated ERK (Santa Cruz Biotechnology, Santa Cruz, CA), phosphorylated IκB (New England BioLabs, Beverly, MA), anti-MyD88, ATG16L1, Beclin-1, Atg5, rabbit anti-LC3 (Cell Signaling, Beverly, MA), or anti-NOD1 and NOD2 (Cayman Chemical, Ann Arbor, MI), and then developed with horseradish peroxidase-conjugated second antibodies (Zymed Laboratories, San Francisco, CA) and enhanced chemiluminescence (Pierce Chemical Co., Rockford, IL). Appropriate exposures to X-ray film were made, and the filters then stripped and re-probed with antibodies to GAPDH (Santa Cruz Biotechnology, Santa Cruz, CA) as appropriate.

### Real-time PCR for mRNA assay

Total RNA was prepared from control or infected cells with the Trizol reagent (Invitrogen Corporation, Carlsbad, CA), following the manufacturer’s directions. The RNA was reverse-transcribed with random hexamers using the GeneAmp kit (Roche, Nutley, NJ) as described in detail earlier [20,21,23,24]. Real-time reverse transcription-PCR analyses were performed in a fluorescence temperature cycler (LightCycler; Roche Diagnostics) as described previously [20,21]. The following primers were used: IL-8, 5′-AAACCACCGGAAGGAACCAT-3′ (forward primer) and 5′-GCCAGCTTGGAAGTCATGT-3′ (reverse primer); hBD-2, 5′-ATCAGCCATGAGGGTCTTGT-3′ (forward primer) and 5′-GAGACCACAGGTGCCAATTT-3′ (reverse primer); or glyceraldehyde-3-phosphate dehydrogenase, 5′-CCAGCCGAGCCACATCGCTC-3′ (forward primer) and 5′-ATGAGCCCCAGCCTTCTCCAT-3’. Standard curves were obtained for each primer set with serial dilutions of cDNA. All quantifications were normalized to the housekeeping gene glyceraldehyde-3-phosphate dehydrogenase. Relative expression was given as a ratio between target gene expression and glyceraldehyde-3-phosphate dehydrogenase expression.

### Small-interfering RNA (siRNA) transfection

All transient transfections were carried out in triplicate using *NeoFX* reagent (Ambion, Austin, TX) to final concentration of 20 nM following the manufacturer’s instructions. The siRNAs used were as follows: NOD1 siRNA and siRNA for the negative control (Invitrogen Corporation, Carlsbad, CA). All siRNA were tested and verified as reducing expression by >80% protein reduction in SW480 cells by immunoblot analysis or reducing expression of >50% of mRNA by real-time PCR when appropriate Ab was not available, as in our previous work [20]. For the SW480 cells, 20nM of each siRNA was transfected 48–96 h before *P. aeruginosa* infection.

### Statistical analysis

All above experiments were carried out at least three times with similar results. Statistical significance was determined using the Student’s *t*-test.

## Results

### Prolonged infection by *P. aeruginosa* resulted in suppression of IL-8 but enhancement of hBD-2 protein secretion

**Figure 1:**
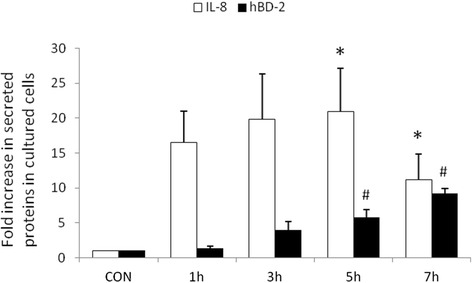
**The effect of prolonged infection by** ***P. aeruginosa*** **on IL-8 and hBD-2 proteins secretion in SW480 cells.** SW480 cells were left uninfected (CON) or infected with the wild-type *P. aeruginosa* strain PAO1 for the times indicated. Supernatant was analyzed by ELISA 6 hours later for IL-8. The amount of IL-8 produced is shown as the fold increase over uninfected control cells. Results are represented as means ± S.E.M. for at least three determinations from independent experiments. (*p <0.05; # *p* <0.05).

The cultured cells were uninfected (control) or infected by *P. aeruginosa* for indicated times. Supernatant of cultured cells was analyzed by ELISA for IL-8 and hBD-2 secretion. As shown in Figure 1, the production of IL-8 and hBD-2 followed two distinct time-courses. First, IL-8 showed a peak after 5 hours and a decline thereafter. A second, distinct time-course was observed for hBD-2, which remained elevated 7 hours after *P. aeruginosa* infection.

### Prolonged infection by *P. aeruginosa* results in enhancement of IL-8 and hBD-2 mRNA expression in SW480 cells

**Figure 2:**
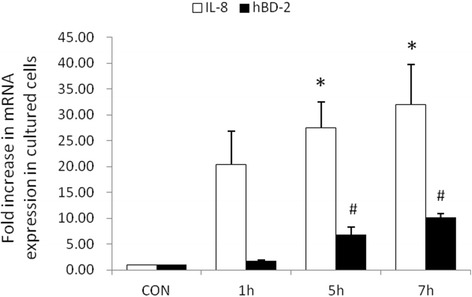
**The effect of prolonged** ***P. aeruginosa*** **infection on IL-8 and hBD-2 mRNA expression in SW480 cells.** SW480 cells were left uninfected (CON) infected with the wild-type *P. aeruginosa* strain PAO1 for one to seven hours. Total RNA was prepared after infection and analyzed by real-time quantitative PCR to estimate amounts of IL-8 and hBD-2 transcript. The amount of IL-8 and hBD-2 mRNA produced, normalized to the corresponding amount of GAPDH transcript, is shown as the fold increase over uninfected control cells. Results are represented as means ± S.E.M. for at least three determinations from independent experiments. (*p <0.05; # *p* <0.05).

We proceeded to examine the effect of *P. aeruginosa* infection on IL-8 and hBD-2 mRNA levels. SW480 cells were either uninfected or infected by *P. aeruginosa*. Total RNA was prepared, reverse transcribed with random hexamers, and analyzed by real-time quantitative PCR. As shown in Figure 2, *P. aeruginosa* infection induced IL-8 mRNA and hBD-2 mRNA expression (normalized to GAPDH) after one-hour infection in SW480 cells. Both IL-8 mRNA and hBD-2 mRNA in SW480 cells continued to increase after prolonged infection.

### The involvement of ERK PI3K/Akt, NF-κB signaling pathway and NOD1 protein in SW480 cells after *P. aeruginosa* infection

**Figure 3:**
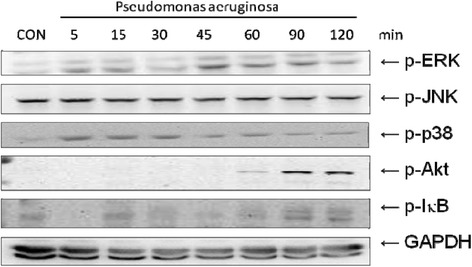
**The proteins expression of intracellular signaling pathways in** ***P. aeruginosa*** **-infected SW480 cells.** SW480 cells were left uninfected (CON), or infected with wild-type *P. aeruginosa* strain PAO1 for the times indicated. Activation of the ERK, JNK, p38, Akt and IκB were analyzed in whole cell protein by immunoblotting with antibodies to phosphorylated (p) ERK, JNK, p38, Akt and IκB. The results shown are representative of three separate experiments. GAPDH worked as a normalization of cytosolic protein.

**Figure 4:**
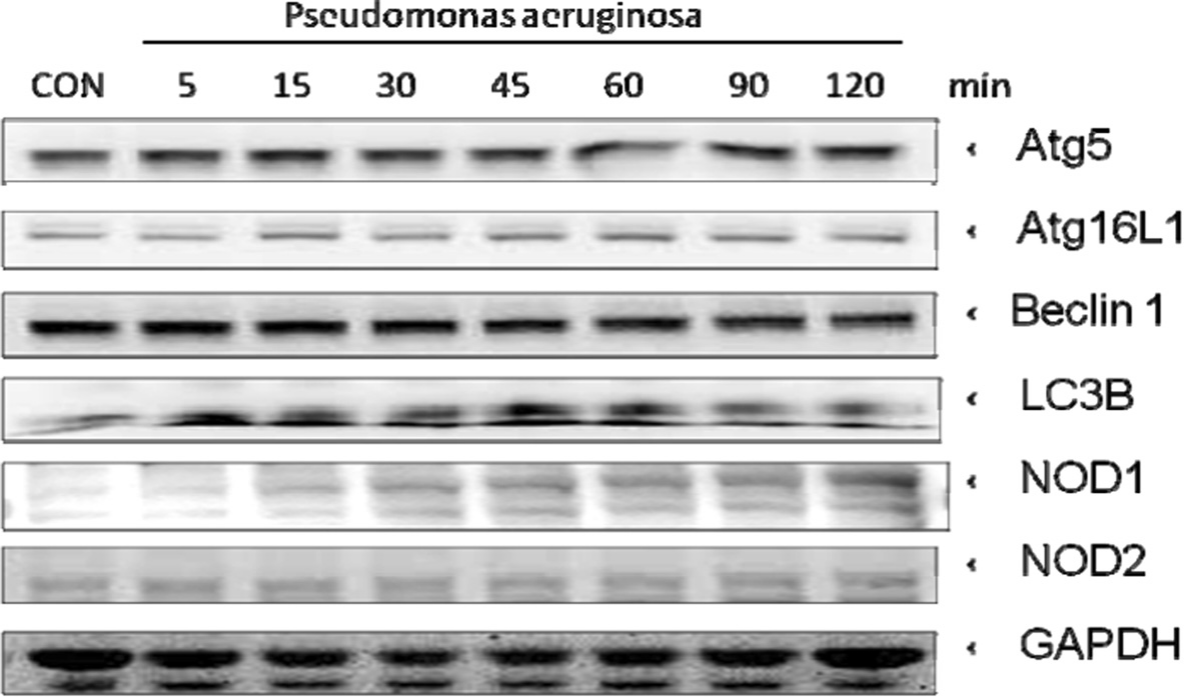
**The proteins expression of autophagy in** ***P. aeruginosa*** **-infected SW480 cells.** SW480 cells were uninfected (CON) or infected by *P. aeruginosa* for indicated times. The Western blots illustrate the expression of Agt5, Atg16L1, Beclin 1, LC3B, NOD1 and NOD2 proteins in cytosolic extracts of SW480 cells infected by *P. aeruginosa* at indicated times. The results shown are representative of three separate experiments. GAPDH worked as a normalization of cytosolic protein.

To further evaluate which signaling pathway is involved in *P. aeruginosa*–induced IL-8 and hBD-2 regulation, we investigated the downstream signal pathways of TLRs and autophagy proteins. SW480 cells were uninfected or infected with PAO1. Activation of the ERK JNK, p38, Akt and IκB, were analyzed in whole cell protein by Western blot. Western blot data showed that protein levels of p-ERK were significantly upregulated at 5 and 15 minutes infection, reached peak at 45 min, and significantly decreased at 120 min when p-Akt and p-IκB were significantly upregulated (Figure 3), suggesting the involvement of ERK, Akt and IκB in the downstream signaling of *P. aeruginosa* infection. However, the activation of JNK or p38 had no significant upregulation from 5 to 120 min. Western blot analysis was also applied to evaluate autophagy proteins expression in *P. aeruginosa*-infected SW480 cells. It was demonstrated that the band density of NOD1 continuously increased after prolonged infection by *P. aeruginosa*, while other autophagy proteins (e.g. NOD2, Beclin1 or Atg5) had the same band density even after prolonged infection (Figure 4). It suggested the involvement of NOD1 in enhanced hBD-2 expression in SW480 cells after prolonged *P. aeruginosa* infection.

### The involvement of ERK and PI3K/Akt signaling pathway in negative regulation of IL-8 after prolonged *P. aeruginosa* infection

**Figure 5:**
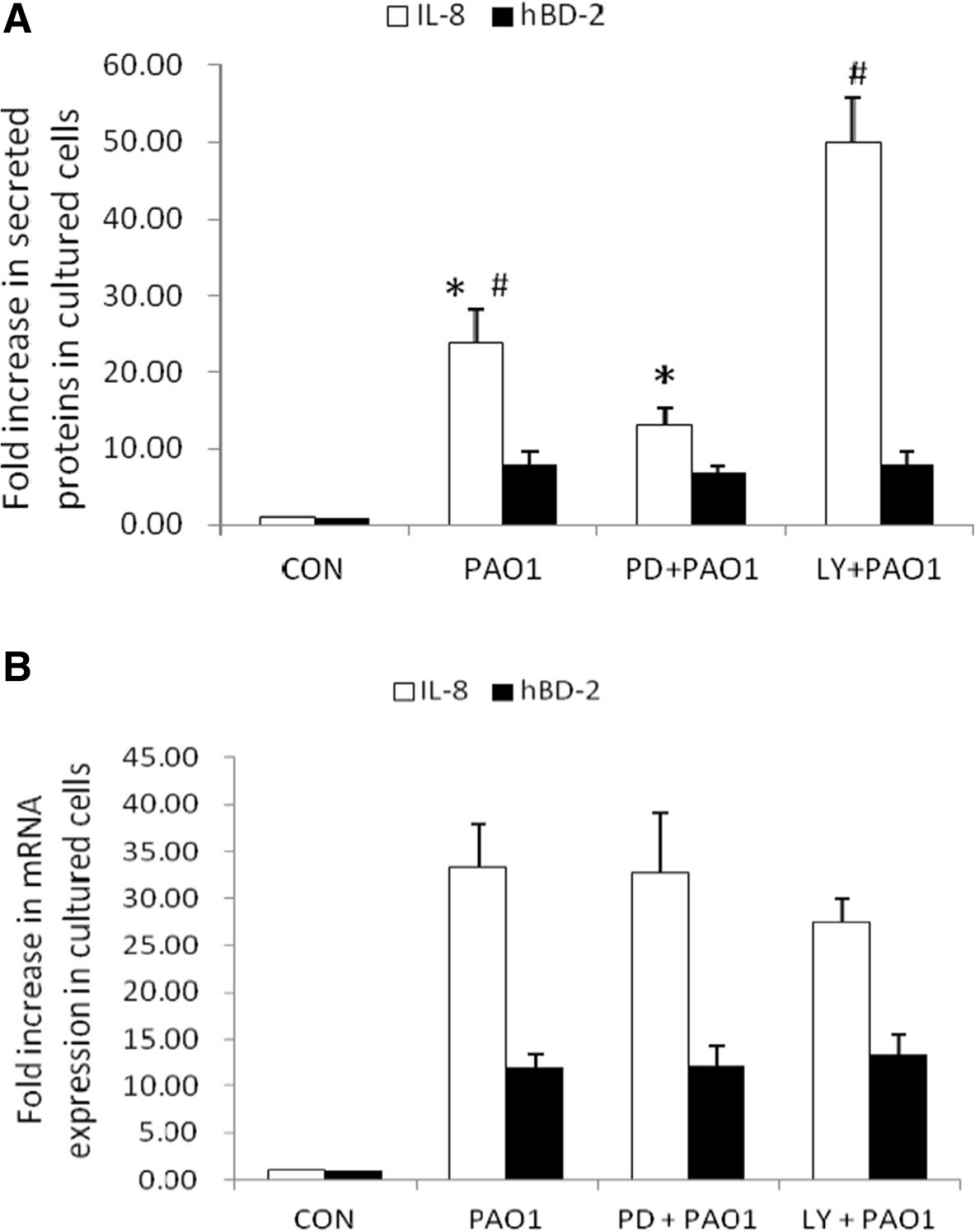
**The involvement of ERK and PI3K/Akt signal pathways in** ***P. aeruginosa*** **-induced IL-8 in SW480 cells. (A)** Effect of ERK and PI3K inhibition on *P. aeruginosa*-induced IL-8 and hBD-2 secretion. SW480 cells were left untreated, or treated with 25 μM PD98059 (PD) and 50 μM LY294002 (LY) for one hour. They were then infected with the wild-type *P. aeruginosa* strain PAO1 for 7 hours. Supernatant was analyzed by ELISA for IL-8 and hBD-2. The amount of IL-8 and hBD-2 produced is shown as the fold increase over uninfected control (CON) cells. **(B)** Effect of ERK and PI3K inhibition on *P. aeruginosa*-induced IL-8 and hBD-2 mRNA. SW480 cells were left untreated, or treated with 25 μM PD98059 (PD) and 50 μM LY294002 (LY). They were then infected with the wild-type *P. aeruginosa* strain PAO1 for 7 hours. Total RNA was prepared and analyzed by real-time quantitative PCR to estimate amounts of IL-8 and hBD-2 transcript. The amount of IL-8 and hBD-2 mRNA produced, normalized to the corresponding amount of GAPDH transcript, is shown as the fold increase over uninfected control (CON) cells. Results are represented as means ± S.E.M. for at least three determinations from independent experiments. (* *p* <0.05; # *p* <0.005).

Pretreatment with ERK and PI3K inhibitors (PD98059 for ERK and LY294002 for PI3K) (Figure 5A) suppressed and upregulated *P. aeruginosa*-induced IL-8 production in SW480 cells respectively. These data indicated that ERK-mediated IL-8 production was suppressed via activation of the PI3K/Akt signaling pathway. We proceeded to examine the involvement of ERK and Akt in the *P. aeruginosa*-induced IL-8 mRNA levels. SW480 cells were either uninfected or infected by *P. aeruginosa* for 7 hours in the absence or presence of PD98059 or LY294002. Total RNA was prepared, reverse transcribed with random hexamers, and analyzed by real-time quantitative PCR. As shown in Figure 5B, *P. aeruginosa* infection induced IL-8 mRNA expression (normalized to GAPDH) in SW480 cells. Both inhibitors had no significant effect on *P. aeruginosa*-induced IL-8 mRNA expression in SW480 cells, suggesting that ERK and PI3K/Akt signal pathways were involved in *P. aeruginosa*-induced IL-8 immune response by post-transcriptional or post-translational mechanisms.

Pretreatment with ERK and PI3K inhibitors (PD98059 for ERK and LY294002 for PI3K) had no significant effect on *P. aeruginosa*-induced hBD-2 production (Figure 5A) or mRNA expression (Figure 5B) in SW480 cells.

### The involvement of NOD1 in positive regulation of hBD-2 after prolonged *P. aeruginosa* infection

**Figure 6:**
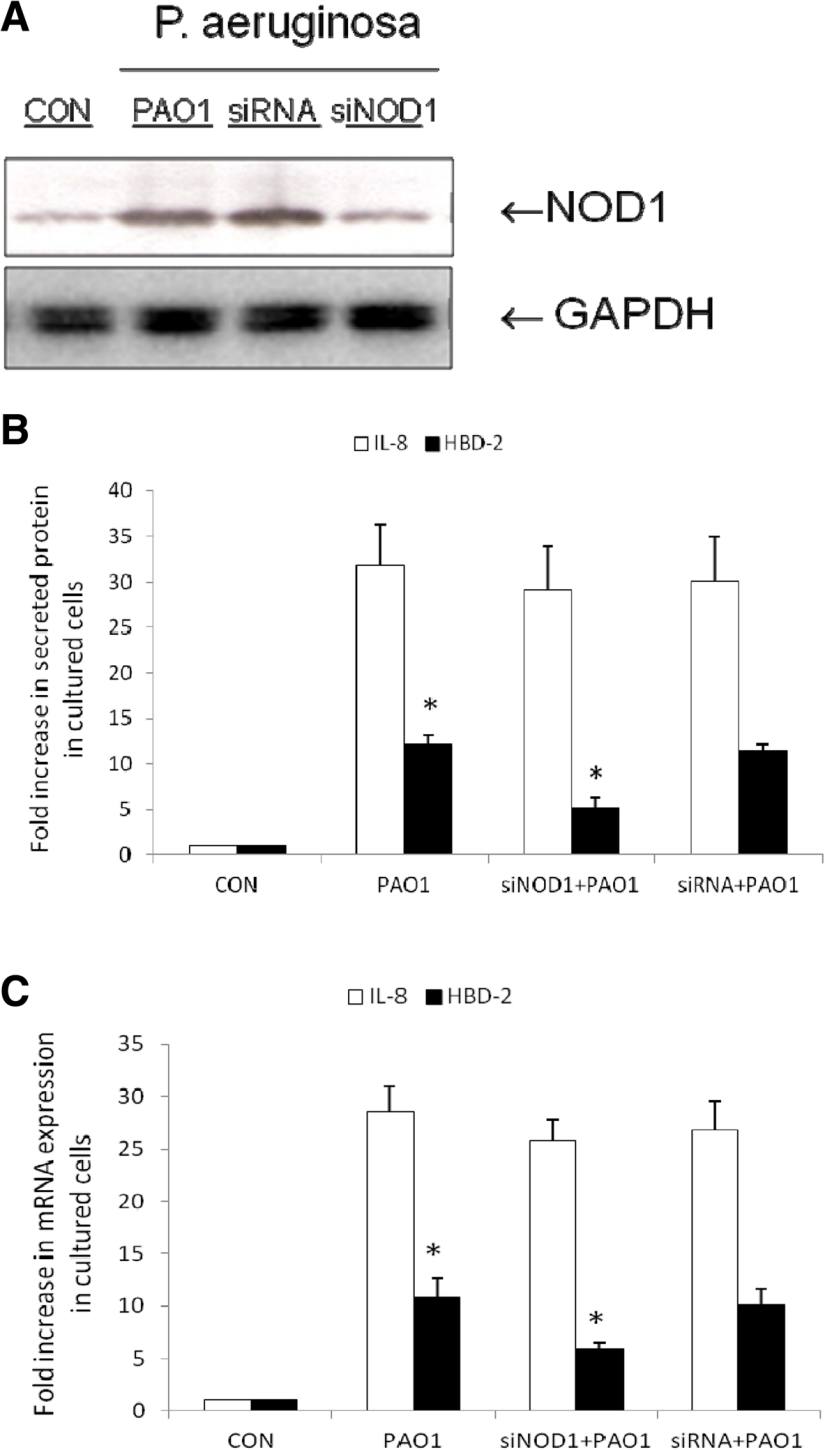
**The involvement of NOD1 in** ***P. aeruginosa*** **-induced hBD-2 in SW480 cells.** SW480 cells were transfected with control siRNA and NOD1 siRNA (siRNA = non-target control siRNA; siNOD1 = siRNA to NOD1) for 48 hours. The transfected cells were left uninfected or infected by *P. aeruginosa* PAO1 for 7 hours. **(A)** Western blots probed with antibodies against NOD1 and GAPDH confirm knockdown of NOD1. **(B)** Supernatant was analyzed by ELISA for secreted IL-8 and hBD-2 protein in SW480 cells. The amount of IL-8 or hBD-2 produced is shown as the fold increase over control cells. **(C)** After infection, total RNA was extracted from the cells and analyzed byReal-time PCR, as described in Materials and Methods. Results are represented as means ± S.E.M. for at least three determinations from independent experiments. An asterisk indicates a significant difference (*p* <0.05).

Based on our observation that the expression of NOD1 protein was upregulated after prolonged *P. aeruginosa* infection, we investigated if NOD1 was involved in the *P. aeruginosa*-induced hBD-2 expression. We adapted a siRNA knockdown approach for NOD1. Knockdown of NOD1 was confirmed by Western blot with specific siRNA in SW480 cells up to 48 hrs (Figure 6A). siRNA-transfected SW480 cells were uninfected or infected by *P. aeruginosa* PAO1 for 7 hours. Following knockdown of NOD1, we detected the *P. aeruginosa*-induced hBD-2 production (Figure 6B) and mRNA expression (Figure 6C) in SW480 cells was diminished in NOD1-silenced cells, but not in control siRNA-silenced cells. It suggested NOD1 was involved in the enhanced hBD-2 expression in SW480 cells by prolonged *P. aeruginosa* infection.

### It is a general phenomenon in Caco-2 intestinal epithelial cell lines

To determine whether the above finding was a general phenomenon among different intestinal epithelial cell lines, the same experiments were undertaken in Caco-2 cells. Caco-2 cells were untreated or pretreated by inhibitors and infected with wild-type *P. aeruginosa* PAO1, using the same experiments as above. The supernatants were analyzed for proteins secretion in Caco-2 cells after treatment of inhibitors or siRNA. The proteins extracted from the cells were analyzed for signaling pathways involved in the regulation. Similar to SW480 cells, we have seen the same results in Caco-2 cells in response to infection (Additional file 1: Figure S1, Additional file 2: Figure S2, Additional file 3: Figure S3 and Additional file 4: Figure S4).

## Discussion

While the expression of antimicrobial molecules in epithelial cells has been shown as a key to control infection, modulation of proinflammatory responses prevents the cells from the detrimental effects of overwhelming inflammation. In several models of bacterial infection in mice, maximum KC (murine IL-8) expression that recruits and activates neutrophils occurs in the first hours, resulting in enhanced clearance of bacteria after *Pseudomonas* challenge [25]. NF-kappa B is a central regulator of the intestinal epithelial cell innate immune response induced by infection with enteroinvasive bacteria [26]. Phosphorylation of IκB (Figure 3), inhibitor of NF-κB, is targeted for proteasome-dependent degradation, leading to translocation of NF-κB to the nucleus and transactivation of NF-κB target genes, giving rise to IL-8 and hBD-2 mRNA expression (Figure 2). However, prolonged production of IL-8 will cause tissue damage. The massive influx of neutrophils into *Pseudomonas*-infected sites is stimulated by an excessive production and release of inflammatory mediators, the most important of which include prolonged and sustained expression of IL-8 [27-29]. The accumulation of neutrophils, a hallmark of inflammation, in turn contributes to tissue destruction [30], particularly at the early stage of *P. aeruginosa* colonization. Consequently, translocation of bacteria and absorption of endotoxins may have profound systemic effects and may result in bacteremia, as well as endotoxemia. Likewise, intensive mucosal injury by massive influx of neutrophils may result in translocation of *P aeruginosa* into blood stream and subsequent sepsis. This can also explain why fever and diarrhea are the two most common initial symptoms in *P. aeruginosa* sepsis in previously healthy infants and children [2].

Substantial evidence has shown that PI3K/Akt activation promotes the internalization of PA strains PAO1 and PAK by epithelial cells [31], and suppresses a proinflammatory response through negatively regulating TLR signaling [32]. Based on our observation that PI3K/Akt pathway plays an anti-inflammatory role, decreasing IL-8 production on *Salmonella* infection in IECs via interactions with ERK kinase [24], we demonstrated that inhibition of PI3K upregulated but inhibition of ERK suppressed *P. aeruginosa*-induced IL-8 production in IECs after prolonged infection (Figure 5). It suggested that the anti-inflammatory PI3K/Akt signal pathway was activated at a late stage to suppress the detrimental effect of IL-8 overexpression after prolonged *P. aeruginosa* infection (Figure 1). In contrast, PI3K/Akt has no significant effect on hBD-2 expression (Figure 5), which is needed to defend the cells against *Pseudomonas* infection continuously. PI3K/Akt signaling pathway promotes host resistance against *P. aeruginosa* infection by suppressing corneal inflammation and perforation in mice [33]. Inhibition of PI3K resulted in worsened disease after *P aeruginosa* corneal infection. This observation is consistent with some studies showing that intraperitoneal injection with wortmannin increased serum cytokines levels and led to susceptibility to sepsis [34]. The pharmaceuticals to block IL-8 or enhance PI3K/Akt signaling pathway may be a promising immunotherapy for antibiotics-resistant *P. aeruginosa,* prevention of *P. aeruginosa* sepsis and lessen usage of antibiotics.

Abnormalities in the handling of intracellular bacteria may allow persistent multiplication of bacteria and trigger chronic uncontrolled intestinal inflammation [35]. A significant increase in the adaptive immunologic response to Crohn’s disease (CD)-associated microbial antigens, such as *Pseudomonas fluorescens*–related protein (I2) and *Escherichia coli* outer membrane porin C (anti-OmpC), is due to the presence of a defective innate immune gene (*NOD2*). HBD-2 is an antimicrobial peptide implicated in the pathogenesis of inflammatory bowel disease (IBD) [36]. Children with CD showed a lower expression of hBD-2 in the inflamed terminal ileum and ascending colon [37]. It suggests that NOD2 mutants result in dysregulation of hBD-2 in mucosa intruded by some *Pseudomonas* species predisposed to IBD. An inappropriate immune response to commensal *Pseudomonas* species is involved in colonic CD etiology [38]. NOD1 or NOD2 prevalence in colon or ileum could also be due to the predominance of different intracellular organisms or enteroinvasive bacteria for which they are receptors. Variants of NOD1 and NOD2 genes display opposite associations with risk of CD [39]. NOD1 is not involved in IBD [40] while NOD2 mutants are susceptible to IBD. It is compatible with the finding that *P. aeruginosa* was only identified in the gut of non-IBD patients [38] because the innate immunity to *P. aeruginosa* is NOD1-dependent (Figures 4 and 6) but not NOD2 (data not shown). NOD1 plays an important role in the initial recognition of pathogenic bacteria at epithelial surfaces, such as the gut, where innate immune responses to commensal bacteria must be avoided [14]. Likewise it was demonstrated that NOD1 but not NOD2, seems to play a role in chronic infection of airway by *P. aeruginosa* in cystic fibrosis patients [41]. Therefore, it is hypothesized that the loss of commensal *P. aeruginosa* in CD patients may result in dysbiosis between *Pseudomonas* species and other gut microbiota, which is directly involved in CD manifestation.

There were many signal pathways reported to be involved in the regulation of hBD-2 in a variety of cultured cells infected by *P. aeruginosa* [33,42,43], including NF-κB, AP-1, PI3K/Akt, p38MAPK, ERK, and JNK. However this is the first time to demonstrate the involvement of NOD1 in *P. aeruginosa*-induced hBD-2 expression in intestinal epithelial cells (Figures 4 and 6). Furthermore, NOD1 expressed by epithelial cells takes part in the activation of NF-κB and the up-regulated production of an important epithelial cell chemoattractant in response to *P. aeruginosa* [44]. Gram-negative bacteria can deliver peptidoglycan to cytosolic NOD1 in host cells via a novel mechanism involving outer membrane vesicles (OMVs) including the Gram-negative mucosal pathogens [45]: *Helicobacter pylori*, *Pseudomonas aeruginosa* and *Neisseria gonorrhoea*. These peptidoglycan-containing OMVs enter epithelial cells through lipid rafts, thereby upregulating NF-κB and inducing NOD1-dependent responses in vitro. Future studies should clarify the role of mucosal NOD1 during *P. aeruginosa* infection in vivo.

## Conclusion

In conclusion, we demonstrated *P. aeruginosa* induced proinflammatory responses and antimicrobial peptide in IECs. Prolonged infection by *P. aeruginosa* results in suppression of IL-8 but enhancement of hBD-2 protein production in SW480 cells, even though both mRNAs were increased gradually. The PI3K/Akt and ERK signaling pathway may be involved in the negative regulation of *P. aeruginosa*-induced IL-8 production in SW480 cells while NOD1 protein was involved in the positive regulation of *P. aeruginosa*-induced hBD-2 expression.

While antimicrobial peptide in epithelial cells has been shown to continuously protect the host against prolonged infection, modulation of proinflammatory responses protects the host from the detrimental effects of overwhelming inflammation. The dysbiosis between *P. aeruginosa* and other *Pseudomonas* species or gut microbiota could be directly involved in the pathogenesis of CD. The differential regulation of NOD1 and NOD2 in intestinal mucosa on the commensal and pathogenic bacteria deserves further investigation.

### Ethics statement

This was an entirely *in-vitro* study that was approved by the Chang Gung University Biosafety Committee.

## Additional files

Additional file 1: Figure S1. The proteins expression of intracellular signaling pathway in *P. aeruginosa*-infected Caco-2 cells. Caco-2 cells were left uninfected (CON), or infected with wild-type *P. aeruginosa* strain PAO1 for the indicated times. Activation of the ERK, Akt, p38 and JNK were analyzed in whole cell protein by immunoblotting with antibodies to phosphorylated (p) ERK, Akt, p38, and JNK. The results shown are representative of three separate experiments. GAPDH worked as a normalization of cytosolic protein. Additional file 2: Figure S2. The proteins expression of autophagy in *P. aeruginosa*-infected Caco-2 cells. Caco-2 cells were uninfected (CON) or infected by wild-type *P. aeruginosa* strain PAO1 for the indicated times. The Western blots illustrate the expression of Agt5, Beclin 1, and NOD1 proteins in cytosolic extracts of Caco-2 cells. The results shown are representative of three separate experiments. GAPDH worked as a normalization of cytosolic protein. Additional file 3: Figure S3. Involvement of ERK and PI3K/Akt signal pathways in *P. aeruginosa*-induced IL-8 and hBD-2 in Caco-2 cells. Effect of ERK and PI3K inhibition on *P. aeruginosa*-induced IL-8 and hBD-2 secretion. Caco-2 cells were left untreated, or treated with 25 μM PD98059 (PD) and 50 μM LY294002 (LY) for one hour. They were then infected with the wild-type *P. aeruginosa* strain PAO1. Supernatant was analyzed by ELISA for IL-8 and hBD-2. The amount of IL-8 or hBD-2 produced is shown as the fold increase over control cells (CON). Results are represented as means ±S.E.M. for at least three determinations from independent experiments. (* *p* < 0.05; # *p* < 0.05). Additional file 4: Figure S4. Involvement of NOD1 in *P. aeruginosa*-induced IL-8 and hBD-2 in Caco-2 cells. Caco-2 cells were transfected with control siRNA and NOD1 siRNA (siRNA = non-target control siRNA; siNod1 = siRNA to NOD1) for 48 hours. The transfected cells were left uninfected or infected by wild-type *P. aeruginosa* strain PAO1. Supernatant was analyzed by ELISA for secreted IL-8 and hBD-2 protein in Caco-2 cells. The amount of IL-8 or hBD-2 produced is shown as the fold increase over control cells (CON). Results are represented as means ±S.E.M. for at least three determinations from independent experiments. An asterisk indicates a significant difference (* *p* < 0.05).
