# Supplementary material for: Differential regulation of interleukin-8 and human beta-defensin 2 in Pseudomonas aeruginosa-infected intestinal epithelial cells
Source: BMC Microbiol. 2014 Nov 30;14:275. doi: 10.1186/s12866-014-0275-6 (PMC4261737; doi:10.1186/s12866-014-0275-6)
Supplement: Additional file 1: Figure S1. — The proteins expression of intracellular signaling pathway in P. aeruginosa-infected Caco-2 cells. Caco-2 cells were left uninfected (CON), or infected with wild-type P. aeruginosa strain PAO1 for the indicated times. Activation of the ERK, Akt, p38 and JNK were analyzed in whole cell protein by immunoblotting with antibodies to phosphorylated (p) ERK, Akt, p38, and JNK. The results shown are representative of three separate experiments. GAPDH worked as a normalization of cytosolic protein. [file 12866_2014_275_MOESM1_ESM.doc]

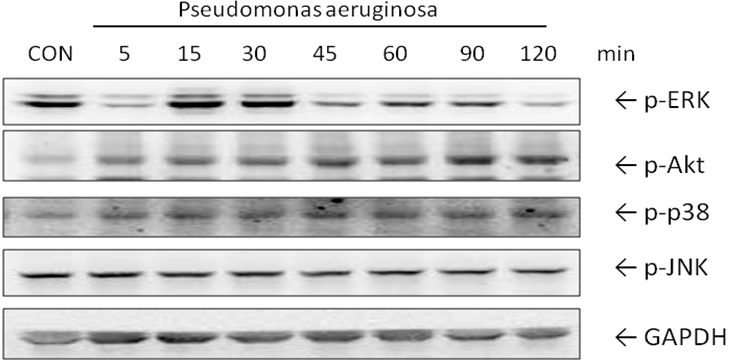
Figure S1

**Figure S1. The proteins expression of intracellular signaling pathway in *P. aeruginosa*-infected Caco-2 cells.** Caco-2 cells were left uninfected (CON), or infected with wild-type *P. aeruginosa* strain PAO1 for the indicated times. Activation of the ERK, Akt, p38 and JNK were analyzed in whole cell protein by immunoblotting with antibodies to phosphorylated (p) ERK, Akt, p38, and JNK. The results shown are representative of three separate experiments. GAPDH worked as a normalization of cytosolic protein.
