# Supplementary material for: Differential regulation of interleukin-8 and human beta-defensin 2 in Pseudomonas aeruginosa-infected intestinal epithelial cells
Source: BMC Microbiol. 2014 Nov 30;14:275. doi: 10.1186/s12866-014-0275-6 (PMC4261737; doi:10.1186/s12866-014-0275-6)
Supplement: Additional file 2: Figure S2. — The proteins expression of autophagy in P. aeruginosa-infected Caco-2 cells. Caco-2 cells were uninfected (CON) or infected by wild-type P. aeruginosa strain PAO1 for the indicated times. The Western blots illustrate the expression of Agt5, Beclin 1, and NOD1 proteins in cytosolic extracts of Caco-2 cells. The results shown are representative of three separate experiments. GAPDH worked as a normalization of cytosolic protein. [file 12866_2014_275_MOESM2_ESM.doc]

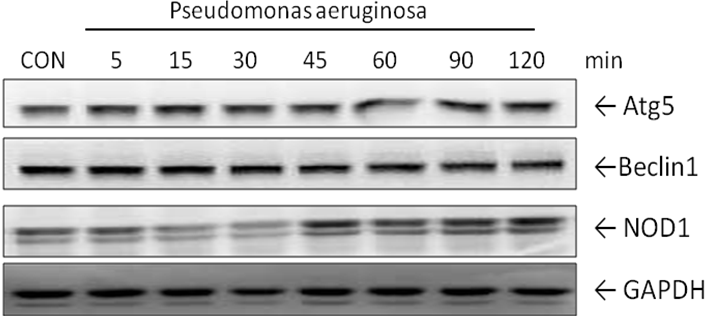
Figure S2

**Figure S2. The proteins expression of autophagy in *P. aeruginosa*-infected Caco-2 cells.** Caco-2 cells were uninfected (CON) or infected by wild-type *P. aeruginosa* strain PAO1 for the indicated times. The Western blots illustrate the expression of Agt5, Beclin 1, and NOD1 proteins in cytosolic extracts of Caco-2 cells. The results shown are representative of three separate experiments. GAPDH worked as a normalization of cytosolic protein.
