# Supplementary material for: Differential regulation of interleukin-8 and human beta-defensin 2 in Pseudomonas aeruginosa-infected intestinal epithelial cells
Source: BMC Microbiol. 2014 Nov 30;14:275. doi: 10.1186/s12866-014-0275-6 (PMC4261737; doi:10.1186/s12866-014-0275-6)
Supplement: Additional file 3: Figure S3. — Involvement of ERK and PI3K/Akt signal pathways in P. aeruginosa-induced IL-8 and hBD-2 in Caco-2 cells. Effect of ERK and PI3K inhibition on P. aeruginosa-induced IL-8 and hBD-2 secretion. Caco-2 cells were left untreated, or treated with 25 μM PD98059 (PD) and 50 μM LY294002 (LY) for one hour. They were then infected with the wild-type P. aeruginosa strain PAO1. Supernatant was analyzed by ELISA for IL-8 and hBD-2. The amount of IL-8 or hBD-2 produced is shown as the fold increase over control cells (CON). Results are represented as means ±S.E.M. for at least three determinations from independent experiments. (* p < 0.05; # p < 0.05). [file 12866_2014_275_MOESM3_ESM.doc]

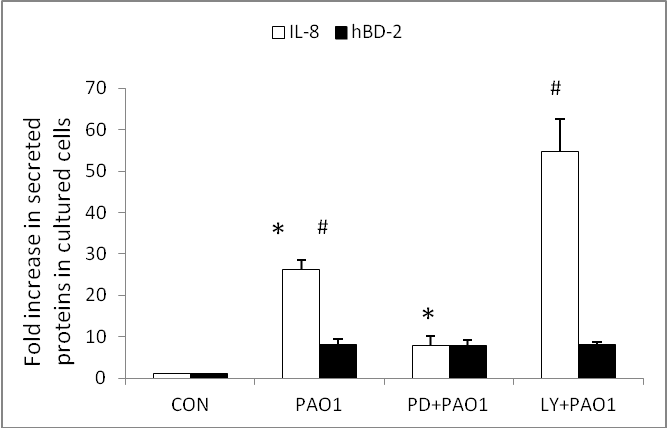
Figure S3

**Figure S3.** **Involvement of ERK and PI3K/Akt signal pathways in *P. aeruginosa*-induced IL-8 and hBD-2 in Caco-2 cells.** Effect of ERK and PI3K inhibition on *P. aeruginosa*-induced IL-8 and hBD-2 secretion. Caco-2 cells were left untreated, or treated with 25 μM PD98059 (PD) and 50 μM LY294002 (LY) for one hour. They were then infected with the wild-type *P. aeruginosa* strain PAO1. Supernatant was analyzed by ELISA for IL-8 and hBD-2. The amount of IL-8 or hBD-2 produced is shown as the fold increase over control cells (CON). Results are represented as means ±S.E.M. for at least three determinations from independent experiments. (* *p* < 0.05; # *p* < 0.05).
