# Supplementary material for: Differential regulation of interleukin-8 and human beta-defensin 2 in Pseudomonas aeruginosa-infected intestinal epithelial cells
Source: BMC Microbiol. 2014 Nov 30;14:275. doi: 10.1186/s12866-014-0275-6 (PMC4261737; doi:10.1186/s12866-014-0275-6)
Supplement: Additional file 4: Figure S4. — Involvement of NOD1 in P. aeruginosa-induced IL-8 and hBD-2 in Caco-2 cells. Caco-2 cells were transfected with control siRNA and NOD1 siRNA (siRNA = non-target control siRNA; siNod1 = siRNA to NOD1) for 48 hours. The transfected cells were left uninfected or infected by wild-type P. aeruginosa strain PAO1. Supernatant was analyzed by ELISA for secreted IL-8 and hBD-2 protein in Caco-2 cells. The amount of IL-8 or hBD-2 produced is shown as the fold increase over control cells (CON). Results are represented as means ±S.E.M. for at least three determinations from independent experiments. An asterisk indicates a significant difference (* p < 0.05). [file 12866_2014_275_MOESM4_ESM.doc]

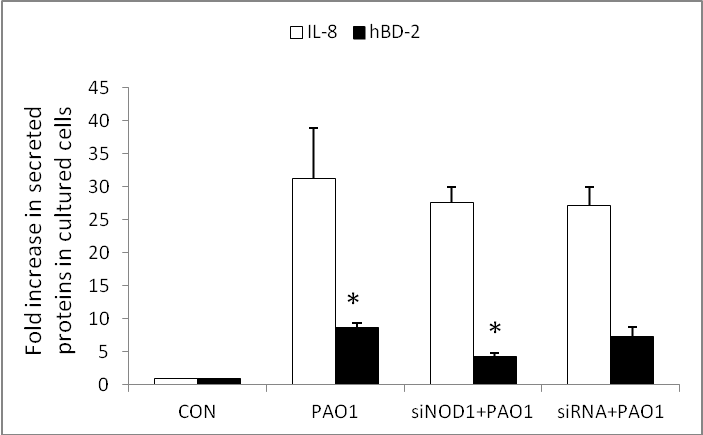
Figure S4

**Figure S4. Involvement of NOD1 in *P. aeruginosa*-induced IL-8 and hBD-2 in Caco-2 cells.** Caco-2 cells were transfected with control siRNA and NOD1 siRNA (siRNA = non-target control siRNA; siNod1 = siRNA to NOD1) for 48 hours. The transfected cells were left uninfected or infected by wild-type *P. aeruginosa* strain PAO1. Supernatant was analyzed by ELISA for secreted IL-8 and hBD-2 protein in Caco-2 cells. The amount of IL-8 or hBD-2 produced is shown as the fold increase over control cells (CON). Results are represented as means ±S.E.M. for at least three determinations from independent experiments. An asterisk indicates a significant difference (* *p* < 0.05).
